# Lower paraoxonase 1 activity in Tunisian bipolar I patients

**DOI:** 10.1186/1744-859X-9-36

**Published:** 2010-10-21

**Authors:** Asma Ezzaher, Dhouha Haj Mouhamed, Anwar Mechri, Manel Araoud, Fadoua Neffati, Wahiba Douki, Lotfi Gaha, Mohamed Fadhel Najjar

**Affiliations:** 1 Laboratory of Biochemistry-Toxicology, Monastir University Hospital, Tunisia; 2 Research Laboratory 'Vulnerability to psychotic disorders LR 05 ES 10', Department of Psychiatry, Monastir University Hospital, Tunisia

## Abstract

**Background:**

The purpose of this study was to investigate the variations of paraoxonase activity and lipid profile in bipolar I patients, and the association of this activity with the sociodemographic, clinical and therapeutic characteristics of this population.

**Patients and methods:**

Our study included 66 patients with bipolar I disorder and 64 controls aged 37.9 ± 12.6 and 36.3 ± 18.2 years, respectively. Paraoxonase activity was determined by kinetic methods; high-density lipoprotein cholesterol (c-HDL), low-density lipoprotein cholesterol (c-LDL), triglycerides and total cholesterol were determined by enzymatic methods; apolipoprotein (Apo)A1, ApoB and lipoprotein (a) (Lp(a)) were determined by immunoturbidimetry using Konelab 30 equipment (Thermo Scientific).

**Results:**

Compared with controls, patients had a significantly lower paraoxonase activity and ApoA1 level, and significantly higher total cholesterol, c-LDL and Lp(a) level and ApoB/ApoA1 ratio. Furthermore, paraoxonase activity was significantly correlated with c-HDL values (r = 0.5612; *P *< 0.001). The lowest paraoxonase activity was noted in relation to age and body mass index (BMI). Moreover, it was associated with gender but not with smoking and alcohol consumption status. In patients, there was no significant change in paraoxonase activity in relation to illness episodes, whereas the lowest values of this activity were seen in manic patients. In contrast, paraoxonase activity was significantly associated with treatment. Indeed, patients taking lithium had the lowest levels.

**Conclusions:**

Bipolar patients had a significant decrease in paraoxonase activity and perturbations in their lipid profile that contribute to increased risk of cardiovascular diseases. Decrease in this activity was significantly associated with treatment with lithium but not with sociodemographic and clinical characteristics. Therefore, such patients require specific care, particularly with regard to their lipid profile.

## Background

Bipolar disorder is a major mood disorder, with an estimated prevalence of about 1% to 3% of the world population, and is characterised by recurrent episodes of depression and mood elevation. It is increasingly recognised that bipolar patients are at higher risk of having chronic general medical conditions such as cardiovascular disease, which is directly associated with increased morbidity and mortality [1]. The exact mechanisms increasing the incidence of cardiovascular risk in bipolar patients remain to be clarified, but they possibly include industrialisation, stress, lack of exercise, dietary lipids (that is, omega-3 fatty acid deficiency) and increasing incidence of smoking and alcohol consumption and other factors [2,3].

Paraoxonase ((PON) (EC 3.1.8.1) aryldialkylphosphatase) is an enzyme synthesised in the liver, mostly located on high-density lipoprotein (HDL) particles, that has been shown to protect or inhibit lipoprotein, which is a key process in the pathophysiology of atherosclerosis [4,5]. PON1 exerts paraoxonase and arylesterase activities as the enzyme hydrolyses organophosphates (such as paraoxon) and aromatic esters (such as phenyl acetate) [6]. Studies investigating the mechanisms underlying the association between bipolar disorder and cardiovascular disease are critical, and there is scant information on the association between this disease and PON1.

This study aims to investigate both the variations of paraoxonase activity and lipid profile in bipolar I patients and the association of this activity with the sociodemographic, clinical and therapeutic characteristics of this population.

## Methods

### Subjects

This study was approved by the local ethical committee and all subjects were of Tunisian origin. Our sample included 66 bipolar I patients from the psychiatry department of the Teaching Hospital of Monastir. The mean age was 37.9 ± 12.6 years, 20 women (37.9 ± 14.3 years) and 46 men (37.9 ± 12.0 years). Consensus on the diagnosis, according to the Diagnostic and Statistical Manual of Mental Disorders, fourth edition (DSM-IV) criteria [7], was made by psychiatrists. The exclusion criteria were age (< 18 years), other psychiatric illnesses, epilepsy or mental retardation. The control group consisted of 64 volunteer subjects without psychiatric or endocrinological diagnoses. The mean age was 36.3 ± 18.2 years, and there were 30 women (41.8 ± 17.3 years) and 34 men (31.4 ± 17.9 years).

All subjects were questioned about their age, gender, previous treatments and cigarette and alcohol consumption habits. The clinical and sociodemographic characteristics are shown in Table 1. Differences between patients and controls for gender, body mass index (BMI) and alcoholic beverage consumption are noted. Therefore, these variables were considered as potential confounder factors for this analysis.

**Table 1:** Sociodemographic and clinical characteristics of the sample population

	Bipolar I patients (n = 66)	Control group (n = 64)	**P **value
Gender, male/female (ratio)	46/20 (2.30)	34/30 (1.13)	0.071

Age, years ± SD	37.9 ± 12.6	36.3 ± 18.2	0.550

BMI, kg/m^2 ^± SD	26.66 ± 4.46	24.88 ± 4.03	0.018

Cigarette smoking			

Smokers	34 (51.5)	28 (43.8)	0.387

Non-smokers	32 (48.5)	36 (56.2)	

Alcoholic beverages			

Consumers	7 (10.6)	14 (21.9)	0.098

Non-consumers	59 (89.4)	50 (78.1)	

Episode of illness			

Depressive (D)	6 (9.1)	-	-

Euthymic (E)	42 (63.6)	-	-

Manic (M)	18 (27.3)	-	-

Treatment:			

Valproic acid	31 (47.0)	-	-

Lithium	5 (6.7)	-	-

Carbamazepine	8 (12.1)	-	-

Antipsychotics	22 (33.3)	-	-

All values are n (%) unless otherwise stated.

BMI = body mass index.

### Samples

After a 12 h overnight fasting, venous blood for each patient was drawn in tubes containing lithium heparinate and immediately centrifuged. The plasma samples were stored at -20°C until the biochemical analysis.

### Biochemical analysis

Paraoxonase activity was determined by kinetic methods using paraoxon as substrate, concentrations of total cholesterol, triglycerides, low-density protein cholesterol (c-LDL) and HDL cholesterol (c-HDL) were determined by enzymatic methods, and apolipoprotein (ApoA1, ApoB) and lipoprotein (a) (Lp(a)) levels were determined by immunoturbidimetric techniques using Konelab 30 equipment (Thermo Electron Corporation, Ruukintie, Finland).

### Clinical evaluations

BMI was calculated as weight (kg) divided by height (m^2^). Obesity was defined when BMI ≥30 kg/m^2 ^[8].

### Statistical analysis

Statistical analyses were performed using SPSS v. 17.0 (SPSS, Chicago, IL, USA). Quantitative variables were presented as mean ± SD and comparisons were performed using the Student's t test. Qualitative variable comparisons were performed using the Χ^2 ^test. Comparisons between patients and controls in paraoxonase activity and lipid profile were performed using analysis of variance (ANOVA) after adjustment for potential confounder factors (gender, BMI and alcoholic beverage consumption). The statistical significance level was set at *P *< 0.05.

## Results

**Figure 1:**
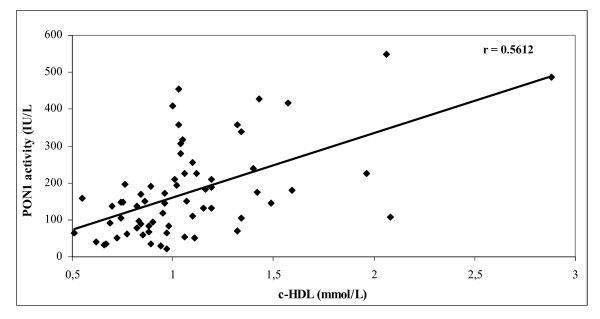
**Correlation between paraoxonase activity and high-density lipoprotein cholesterol (c-HDL) values**.

Compared with control subjects, bipolar I patients had significantly lower paraoxonase activity and ApoA1 levels, and significantly higher values for total cholesterol, c-LDL, ApoB, ApoB/ApoA1 ratio and Lp(a) (Table 2). Furthermore, paraoxonase activity was significantly correlated with c-HDL values (r = 0.5612; *P *< 0.001) (Figure 1).

**Table 2:** Comparisons of biological variables between bipolar I patients and control group

Biological variables	Patients (n = 66)	Controls (n = 64)	P value	P value*
Paraoxonase activity, IU/L	173 ± 123	263 ± 92	< 0.001	< 0.001

Cholesterol, mmol/L	4.41 ± 1.03	3.65 ± 0.80	< 0.001	< 0.001

c-HDL, mmol/L	1.07 ± 0.34	1.07 ± 0.22	0.98	0.426

c-LDL, mmol/L	2.44 ± 1.14	1.22 ± 0.66	< 0.001	< 0.001

Triglycerides, mmol/L	1.75 ± 1.52	1.53 ± 0.94	0.29	0.746

ApoA1, g/L	1.23 ± 0.26	1.42 ± 0.35	< 0.001	0.002

ApoB, g/L	0.85 ± 0.27	0.75 ± 0.26	0.02	0.07

ApoB/ApoA1	0.71 ± 0.23	0.54 ± 0.24	< 0.001	< 0.001

Lipoprotein (a), mg/L	243 ± 223	156 ± 120	< 0.001	< 0.001

*ANOVA test adjusted by gender, BMI and alcoholic beverage.

Apo = apolipoprotein; BMI = body mass index; c-HDL = high-density lipoprotein cholesterol; c-LDL = low-density lipoprotein cholesterol.

After adjustment of paraoxonase activity and lipid profile for confounder factors, we noted a significant association between bipolar disorder and low paraoxonase activity (*P *< 0.0001), low ApoA1 values (*P *< 0.0001), hypercholesterolaemia (*P *< 0.0001), high c-LDL values (*P *< 0.0001), high ApoB/ApoA1 ratio (*P *< 0.0001) and high Lp(a) values (*P *< 0.0001), but no significant association was observed with ApoB (*P *= 0.07) values (Table 3).

**Table 3:** Comparisons of paraoxonase activity between patients and control group by demographic and clinical variables

Variables	Bipolar I patients (n = 66)		Control subjects (n = 64)		P value
		
	N	Mean ± SD	N	Mean ± SD	
Gender					

Male	46	170 ± 125	34	232 ± 95	0.01

Female	20	180 ± 121	30	298 ± 75*	< 0.001

Age, years					

< 25	11	124 ± 82	24	254 ± 87	< 0.001

25-60	51	189 ± 131	32	266 ± 96	0.003

≥60	4	107 ± 51	8	274 ± 97	0.003

BMI, kg/m^2^					

< 25	28	162 ± 119	39	257 ± 101	0.001

25-30	22	186 ± 135	16	284 ± 86	0.008

≥30	16	175 ± 117	9	251 ± 52	0.04

Cigarette smoking					

Yes	34	180 ± 122	28	235 ± 92	0.04

No	32	167 ± 125	36	284 ± 87*	< 0.001

Alcoholic beverages					

Yes	7	236 ± 121	14	243 ± 102	0.89

No	59	166 ± 122	50	268 ± 89	2.10 × 10^6^

Episode of illness					

Depressive	6	254 ± 174			

Euthymic	42	177 ± 123			

Manic	18	137 ± 94			

Treatment					

Lithium (A)	5	73 ± 45			

Carbamazepine (B)	8	175 ± 114			

Valproic acid	31	180 ± 121			

All mood stabilisers	44	167 ± 117			

Antipsychotics	22	187 ± 137			

*A vs B: *P *< 0.05.

BMI = body mass index.

We found that paraoxonase activity was significantly lower in both male and female patients compared to control subjects of the same gender. Moreover, in patients, this parameter was less in men than women.

The lowest paraoxonase activity was noted in patients compared to controls regardless of age and BMI (Table 3). In patients, the least paraoxonase activity was found in subjects with BMI values below 25 kg/m^2^; and those aged more than 60 years (Table 3).

Paraoxonase activity was significantly lower in both smoking and non-smoking patients compared to controls with the same smoking status. In patients, non-smokers had lower values of this parameter than smokers (Table 3).

With regard to alcohol consumption status, for non-consumers patients had significantly lower values of paraoxonase activity than controls. Moreover, they had lower values than patients who consumed alcohol (Table 3).

In patients, there was no significant change in paraoxonase activity in relation to illness episodes. However, manic patients had lower mean values of paraoxonase activity than others (Table 3). We also showed that this activity was significantly associated with the treatment. Indeed, patients taking lithium had the lowest levels of this parameter (Table 3).

## Discussion

Bipolar patients had significantly lower paraoxonase activity than control subjects. These alterations in the plasma paraoxonase activity levels could be one of the missing factors in understanding the relationship between psychiatric disorders and increased cardiovascular risk. In fact, some studies have reported that psychiatric disorders, particularly bipolar disorder, are significantly associated with adverse cardiovascular events and coronary heart disease [9].

The significantly lower paraoxonase activity was noted in patients compared with controls regardless of age and BMI. This may confirm the effect of bipolar disorder on this parameter. The mechanism is not clear, but it has been reported that PON1, which is located on HDL, plays a role in protection against oxidative modification of LDL (that is, lipid peroxidation). Oxidised LDL (oxLDL) is capable of causing neurocytotoxicity. It has been reported that the presence of oxLDL results in the degeneration of neurons, and that the neurocytotoxicity of oxLDL can be attenuated by pretreatment with antioxidants [10]. Patients had significantly higher levels of total cholesterol, c-LDL, ApoB/ApoA1 ratio and Lp(a), and significantly lower levels of ApoA1 than control subjects. The underlying mechanism for the altered lipid status in bipolar patients is unclear. A possible explanation might be found in the patient's nutritional status, the decrease in physical activity and the medications used. Chung *et al*. [11] reported that bipolar disorder is associated with perturbations in lipid profile, which plays an important role in the pathophysiology of mood disorders and particularly in bipolar disorders. Additionally, cholesterol is one component of circulating lipoprotein particles that, besides handling cholesterol, carries micronutrients such as vitamins A and E as well as triglycerides and phospholipids. The latter compounds give rise to substrates such as fatty acids and choline, which are used in both the structural lipids of neuronal membranes and intercellular communication. Therefore, higher levels of one or more compounds of lipoprotein particles circulating in the bloodstream may produce subtle but measurable enhancements of mental processes by influencing the supply of fat-soluble micronutrients, specific fatty acids, or structural lipids [12].

We showed a significant positive correlation between paraoxonase activity and c-HDL values. Paraoxonase is a calcium-dependent esterase closely associated with the high-density lipoprotein subfraction that contains apolipoprotein AI but not apolipoprotein AII in human serum. Previous studies have suggested that HDL can prevent oxidation of LDL and that some oxidised LDL phospholipids are physiological substrates for serum PON1 [13].

In patients, the lowest paraoxonase activity was found in men. This can also be attributed to the protective effect of oestrogen against cardiovascular disease in premenopausal women [2].

Our study failed to show any significant association between paraoxonase activity and cigarette smoking, while non-smoking patients were found to have the lowest levels of this activity. This finding is not in agreement with the studies of Nishio *et al*. [14].

Among alcoholic beverage consumers, we found that patients who consumed alcohol had higher paraoxonase activity levels than control subjects. This could confirm the beneficial effects of moderate consumption of alcoholic beverages with regard to this parameter. Indeed, in our study, all patients who consumed alcohol (n = 7) did so in moderation. According to Sierksma *et al*. [15], moderate alcohol consumption has been found to be associated with slight increases in PON1 activity and HDL cholesterol in normal volunteers.

We noted that there was no significant change in paraoxonase activity in relation to illness episodes; however, manic patients had the lowest mean values of this activity. This may explain the high risk of cardiovascular disease in manic patients compared with depressive patients [16]. Additionally, Angst *et al*. [17] showed that individuals with bipolar I disorder are at greater risk for cardiovascular mortality than individuals with bipolar II disorder. However, the difference in cardiovascular mortality between the two bipolar subtypes reflects the manic symptom burden, which predicts cardiovascular mortality independently of diagnosis and cardiovascular risk factors at intake. The results suggest that mania, either directly (through factors intrinsic to illness) or indirectly (through other mediators or associated variables), may itself influence cardiovascular disease.

We demonstrated that this activity was associated with treatment. Indeed, patients taking lithium had the lowest levels for this parameter. The intimate correlation found between c-HDL and PON1 and the significant negative effect of lithium on c-HDL values reported by Ani *et al*. [18] may explain the significant decrease in paraoxonase activity mean values in patients treated with the same drug.

## Limitations

Several methodological limitations should be considered when interpreting these findings. First, a larger sample size of groups would be beneficial. Second, our work is a cross-sectional study that does not permit follow-up of biological parameters. Finally, the sample of bipolar patients may not be representative of more heterogeneous populations.

## Conclusions

Bipolar patients had significant decreases in paraoxonase activity and perturbations in their lipid profiles, contributing to increased risk of cardiovascular disease. This decrease was independent of age and BMI. Moreover, it was associated with gender but not with smoking and alcohol consumption status.

Paraoxonase activity was not significantly associated with illness episodes. However, the lowest values for this were found in manic patients. In contrast, paraoxonase activity was associated with treatment. Indeed, patients taking lithium had the lowest levels for this parameter. Therefore, such patients should undergo regular lipid profile testing as well as body weight checks. Clinicians should track the effects of the treatment on physical and the biological parameters, and should facilitate access to appropriate medical care.

## Competing interests

The authors declare that they have no competing interests.

## Authors' contributions

AE conceived the study, gathered and managed the data, carried out the immunoassays, performed the literature search and statistical analysis and wrote the paper. DHM participated in the literature search, the management of the data, statistical analysis and writing of the paper. AM contributed to the clinical and rating evaluations during the follow-up periods and participated in the statistical analysis. FN and AM participated in carrying out the immunoassays. WD participated in designing the study, analysing the data, writing the paper and in correction of the final manuscript. LG contributed to the clinical and rating evaluations during the follow-up periods. MFN participated in designing the study, analysing the data, writing the paper and in correction of the final manuscript. All authors read and approved the final manuscript.
